# Fast-track surgery versus traditional perioperative care in laparoscopic colorectal cancer surgery: a meta-analysis

**DOI:** 10.1186/1471-2407-14-607

**Published:** 2014-08-23

**Authors:** Jun-hua Zhao, Jing-xu Sun, Peng Gao, Xiao-wan Chen, Yong-xi Song, Xuan-zhang Huang, Hui-mian Xu, Zhen-ning Wang

**Affiliations:** Department of Surgical Oncology and General Surgery, the First Hospital of China Medical University, Shenyang, 110001 People’s Republic of China

**Keywords:** Fast track surgery, Laparoscopic surgery, Colorectal cancer

## Abstract

**Background:**

Both laparoscopic and fast-track surgery (FTS) have shown some advantages in colorectal surgery. However, the effectiveness of using both methods together is unclear. We performed this meta-analysis to compare the effects of FTS with those of traditional perioperative care in laparoscopic colorectal cancer surgery.

**Methods:**

We searched the PubMed, EMBASE, Cochrane Library, and Ovid databases for eligible studies until April 2014. The main end points were the duration of the postoperative hospital stay, time to first flatus after surgery, time of first bowel movement, total postoperative complication rate, readmission rate, and mortality.

**Results:**

Five randomized controlled trials and 5 clinical controlled trials with 1,317 patients were eligible for analysis. The duration of the postoperative hospital stay (weighted mean difference [WMD], –1.64 days; 95% confidence interval [CI], –2.25 to –1.03; p < 0.001), time to first flatus (WMD, –0.40 day; 95% CI, –0.77 to –0.04; p = 0.03), time of first bowel movement (WMD, –0.98 day; 95% CI, –1.45 to –0.52; p < 0.001), and total postoperative complication rate (risk ratio [RR], 0.67; 95% CI, 0.56–0.80; p < 0.001) were significantly reduced in the FTS group. No significant differences were noted in the readmission rate (RR, 0.64; 95% CI, 0.41–1.01; p = 0.06) or mortality (RR, 1.55; 95% CI, 0.42–5.71; p = 0.51).

**Conclusion:**

Among patients undergoing laparoscopic colorectal cancer surgery, FTS is associated with a significantly shorter postoperative hospital stay, more rapid postoperative recovery, and, notably, greater safety than is expected from traditional care.

## Background

Colorectal cancer is the third most commonly diagnosed cancer in men and the second most commonly diagnosed cancer in women [1]. Surgery, which is still the most common treatment for colorectal cancer, remains a high-risk procedure with clinically significant postoperative stress, complications, and a lengthy postoperative hospital stay. Standard elective colorectal resection is associated with a complication rate of 8% to 20% and a postoperative stay of 8 to 12 days [2]. The high complication rate and long hospital stay necessitate changes to the management of colorectal cancer.

Laparoscopy for colorectal surgery was first reported in 1991 by Fowler [3]. Many studies have shown that this technique can result in a shorter postoperative hospital stay, a lower requirement for postoperative pain control, and more rapid gastrointestinal recovery than can open surgery, without comprising safety [4, 5]. Fast-track surgery (FTS), also termed an enhanced recovery program, was initiated by the Kehlet group in 2001 [6, 7]. This program combines several methods, such as patient education, epidural or regional anesthesia, minimally invasive techniques, no routine use of drains or nasogastric tubes, optimal pain control, and early enteral nutrition and ambulation [6]. Its purpose is to reduce the stress response, shorten the hospital stay, improve recovery, and reduce the complication rate [2]. Many randomized controlled trials (RCTs) and meta-analyses have demonstrated that FTS is applicable and effective in colorectal surgery [8–11].

Indeed, both the laparoscopic technique and FTS are able to enhance recovery and shorten the postoperative hospital stay. Hypothetically, we can assume that incorporation of FTS into laparoscopic surgery can result in the most rapid postoperative recovery. However, this theory is not evidenced-based because very few published comprehensive systematic reviews or meta-analyses on the enhanced recovery effects of FTS in patients undergoing laparoscopic colorectal surgery have been retrieved from the databases. At the same time, well-designed comprehensive studies to provide solid evidence for further studies are needed [12, 13]. Moreover, the individual studies that have investigated this issue have yielded conflicting results. Thus, we conducted the present meta-analysis of published studies to evaluate the effects of FTS in patients undergoing laparoscopic colorectal cancer surgery.

## Methods

### Search strategy

Publications were identified by searching major medical databases, including PubMed, EMBASE, the Cochrane Library, and Ovid, for all articles published until 1 April 2014. We used the following key words: “fast track”, “multimodal rehabilitation”, “enhanced recovery”, “colorectal surgery”, “colorectal resection”, “large intestine”, “colon”, “rectum”, “sigmoid”, “minimally invasive surgery”, and “laparoscopic”. We then broadened the search range by browsing the related summary, methods, and reference sections of retrieved articles. The language used in publications was restricted to English.

### Inclusion and exclusion criteria

Studies that met the following criteria were included: (1) publications in English comparing FTS with conventional perioperative care in patients undergoing laparoscopic colorectal cancer surgery, (2) full text of the article available with a clear description of the FTS protocol used in the study, and (3) reporting of at least one of the outcome measures mentioned below. If overlap between authors or centers was present, the higher-quality or more recent study was selected. Studies were excluded for the following reasons: FTS and traditional perioperative care were not compared or patients with benign colorectal disease were included, or the study did not provide an FTS protocol or the protocol applied fewer than six fast-track elements.

### Outcome measures, data extraction, and assessment of risk of bias

The primary outcomes included the duration of the postoperative hospital stay, time to first flatus, and time of first bowel movement, each measured in days. We also included the total postoperative complication rate (complications defined based on the Memorial Sloan–Kettering Cancer Center complication reporting system [14]), readmission rate, and 30-day postoperative mortality rate. Two authors independently extracted the data from the full text articles using a unified data sheet. The RCTs were evaluated using the Jadad composite scale. High-quality trials were those that scored ≥3 of a maximum possible score of 5. The controlled clinical trials were evaluated using the Newcastle–Ottawa Scale. High-quality trials were those that scored ≥7 of a maximum possible score of 9. Moderate-quality trials scored ≥5. Any disagreement was presented to a third author and resolved by discussion among the investigators.

### Statistical analysis

This meta-analysis was conducted with Review Manager software (RevMan version 5.2; Cochrane Collaboration). The risk ratio (RR) was used for statistical analysis of dichotomous variables, and the weighted mean difference (WMD) was used to analyze continuous variables. Both were reported with 95% confidence intervals (CIs). For continuous variables, if the study provided medians and ranges instead of means and standard deviations, we calculated the means and standard deviations according to the methods provided by Hozo et al. [15]. If the median and interquartile range were provided, the median was used as the mean and the interquartile range divided by 1.35 was used as the standard deviation as described in the Cochrane handbook. And subgroup analysis was performed based on study design and each FT element. Heterogeneity was determined using the χ^2^ test or Cochran Q statistic, and I^2^ was used to quantify heterogeneity. A p value of <0.10 with an I^2^ value of >50% was indicative of substantial heterogeneity. The inverse variance method with a fixed-effects model was applied if no heterogeneity was considered, whereas a random-effects model was used in opposite cases. Publication bias was tested using a funnel plot. The p value threshold for statistical significance was set at 0.05.

## Results

### Eligible studies

**Figure 1:**
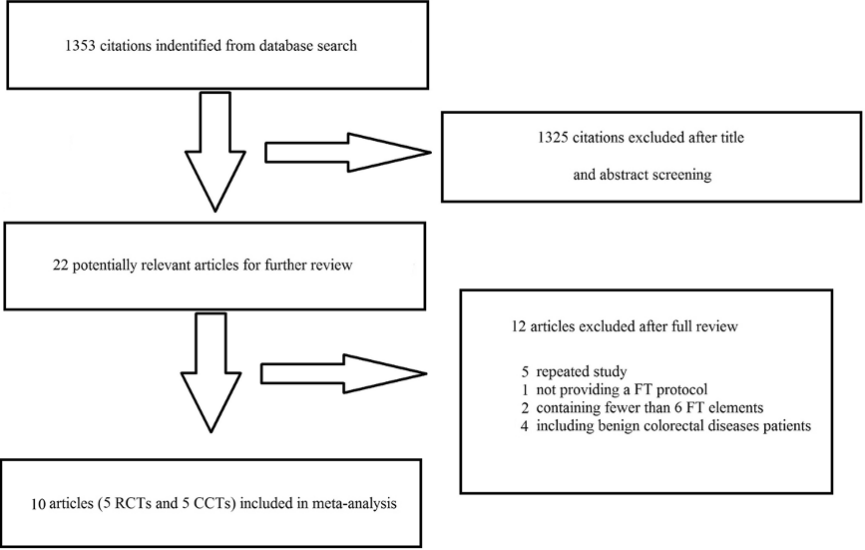
**Flow chart of articles selection.**

By searching the above-mentioned key words, 1,353 citations were identified. Five RCTs [16–20] and five CCTs [21–25] were considered eligible for the meta-analysis (Figure 1). Analysis was performed on 1,317 patients in the FTS group (n = 696) or traditional care group (n = 621). Detailed patient characteristics are listed in Table 1. The included studies had a clearly defined FTS protocol, which included at least six fast-track elements. The detailed information on the fast-track elements included in each study is listed in Table 2. All five RCTs had Jadad scores of ≥3 and were thus considered to be high-quality studies (Table 3). All of the CCTs scored 6 on the Newcastle–Ottawa Scale and were thus considered to be moderate-quality studies (Table 4).

**Table 1:** **Main characteristics of including studies**

Reference	Year	Place	Type	Number of patients		Follow-up	Age Mean ± SD/median (range)		Sex (male/female)		ASA				TNM stage*			
											FT		TC		FT		TC	
				FT	TC		FT	TC	FT	TC	I/II	III/IV	I/II	III/IV	≤stage II	>stage II	≤stage II	>stage II
Lee [19]	2011	Korea	RCT	46	54	1 month	61.9 ± 11.2	60.6 ± 10.0	26/20	30/24	43	2	51	3	23	21	31	21
Vlug [18]	2011	Netherlands	RCT	100	109	30 days	66 ± 8.6	68 ± 8.8	53/47	68/41	82	21	87	22	NA	NA	NA	NA
Q.Wang [16]	2012	China	RCT	40	38	More than one month	71(65-81)	72(65-82)	22/18	20/18	NA	NA	NA	NA	18	22	18	20
G.Wang [17]	2012	China	RCT	40	40	30 days	55.7 ± 17.3	56.1 ± 14.6	27/13	26/14	33	7	36	4	24	16	27	13
Feng [20]	2014	China	RCT	57	59	4 weeks	54.0 ± 12.0	56.3 ± 11.5	36/21	40/19	57	0	59	0	35	22	30	29
Esteban [23]	2014	Spain	CCT	150	56	30 days	68.04 ± 9.9	64.8 ± 14	70/80	28/28	99	49	44	11	NA	NA	NA	NA
Gouvas [21]	2012	Greece	CCT	42	33	1 month	64(31-83)	68(34-85)	22/20	11/22	37	5	29	4	35	7	28	5
Poon [22]	2010	Chinese HongKong	CCT	96	84	Till discharge	72(31-94)	72(46-92)	51/45	50/34	83	13	68	16	54	42	43	41
Vassiliki [24]	2009	USA	CCT	82	115	Till discharge	68.2 ± 13.4	69.3 ± 11.9	36/46	60/55	56	26	76	39	NA	NA	NA	NA
Huibers [25]	2012	Netherlands	CCT	43	33	Till discharge	66(36-79)	64(27-88)	27/16	22/11	33	10	26	7	23	20	26	7

FT: fast track; TC: traditional care; RCT: randomized controlled trails; CCT: clinical controlled trails.

*: the study by Lee included a few submucosal lipoma and lymphoma patients that cannot be staged by TNM.

**Table 2:** **Details about fast track elements of including studies**

Reference	Type	Preoperative					Perioperative					Postoperative							Total
		A	B	C	D	E	F	G	H	I	J	K	L	M	N	O	P	Q	
Lee [19]	RCT	√				√						√		√	√		√	√	**7**
Vlug [18]	RCT	√	√	√	√	√		√	√	√	√	√	√	√	√	√	√	√	**16**
Q.Wang [16]	RCT	√	√	√	√	√			√					√	√		√	√	**10**
G.Wang [17]	RCT	√	√	√		√			√			√	√	√	√			√	**10**
Feng [20]	RCT		√	√				√		√			√	√	√	√		√	**9**
Esteban [23]	CCT	√	√	√		√	√	√	√			√	√	√	√		√	√	**13**
Gouvas [21]	CCT	√	√			√	√	√	√			√		√	√				**9**
Poon [22]	CCT	√				√				√		√	√	√	√			√	**8**
Vassiliki [24]	CCT	√		√										√	√	√		√	**6**
Huibers [25]	CCT	√	√	√	√	√	√	√	√			√		√	√	√	√	√	**14**

RCT: randomized controlled trails; CCT: clinical controlled trails.

A: patients education B: preoperative feeding C: No bowel preparation D: No premedication E: fluid restriction F: high O2 concentration during operation G: prevention of hypothermia during surgery H: epidural analgesia I: wound infiltration with local analgesia J: minimally invasive incisions K: No routine use of NG tube L: No routine use of drains M: early mobilization N: enforced early postoperative oral feeding O: No morphine use P: standard laxatives Q: early remove bladder catheter.

**Table 3:** **The risk of bias of RCTS (Jadad scale)**

Reference	Randomization	Blinding	Withdraw and dropout	Jadad’s score	Quality
Lee [19]	2	0	1	3	High
Vlug [18]	2	1	1	4	High
Q.Wang [16]	2	0	1	3	High
G.Wang [17]	2	0	1	3	High
Feng [20]	2	1	1	4	High

Randomization: randomization was described with appropriate method: 2 score, randomization was described without appropriate method: 1 score, no randomization: 0 score.

Blinding: blinding was performed on all doctors and patients: 2 score, blinding was partially performed on doctors and patients: 1 score, no blinding: 0 score;

Withdraw and dropout: the reason of withdraw and dropout was described: 1 score, the reason of withdraw and dropout was not described: 0 score.

Quality: High-quality trials should score ≥ 3.

**Table 4:** **The risk of bias of RCTS (NOS)**

Reference	Selection				Comparability		Outcome			TOTAL	Quality
	REC	SNEC	AE	DO	SC	AF	AO	FU	FUO		
Esteban [23]	1	0	1	1	0	0	1	1	1	6	Moderate
Gouvas [21]	1	0	1	1	0	0	1	1	1	6	Moderate
Poon [22]	1	1	1	1	0	0	1	0	1	6	Moderate
Vassiliki [24]	1	1	1	1	0	0	1	0	1	6	Moderate
Huibers [25]	1	1	1	1	0	0	1	0	1	6	Moderate

REC: representativeness of the exposed cohort; SNEC: selection of the non-exposed cohort; AE: ascertainment of exposure; DO: demonstration that outcome of interest was not present at start of study; SC: study controls for age, sex; AF: study controls for any additional factors; AO: assessment of outcome; FU: follow-up long enough for outcomes to occur; FUO: adequacy of follow-up of cohorts.

### Duration of postoperative hospital stay

**Figure 2:**
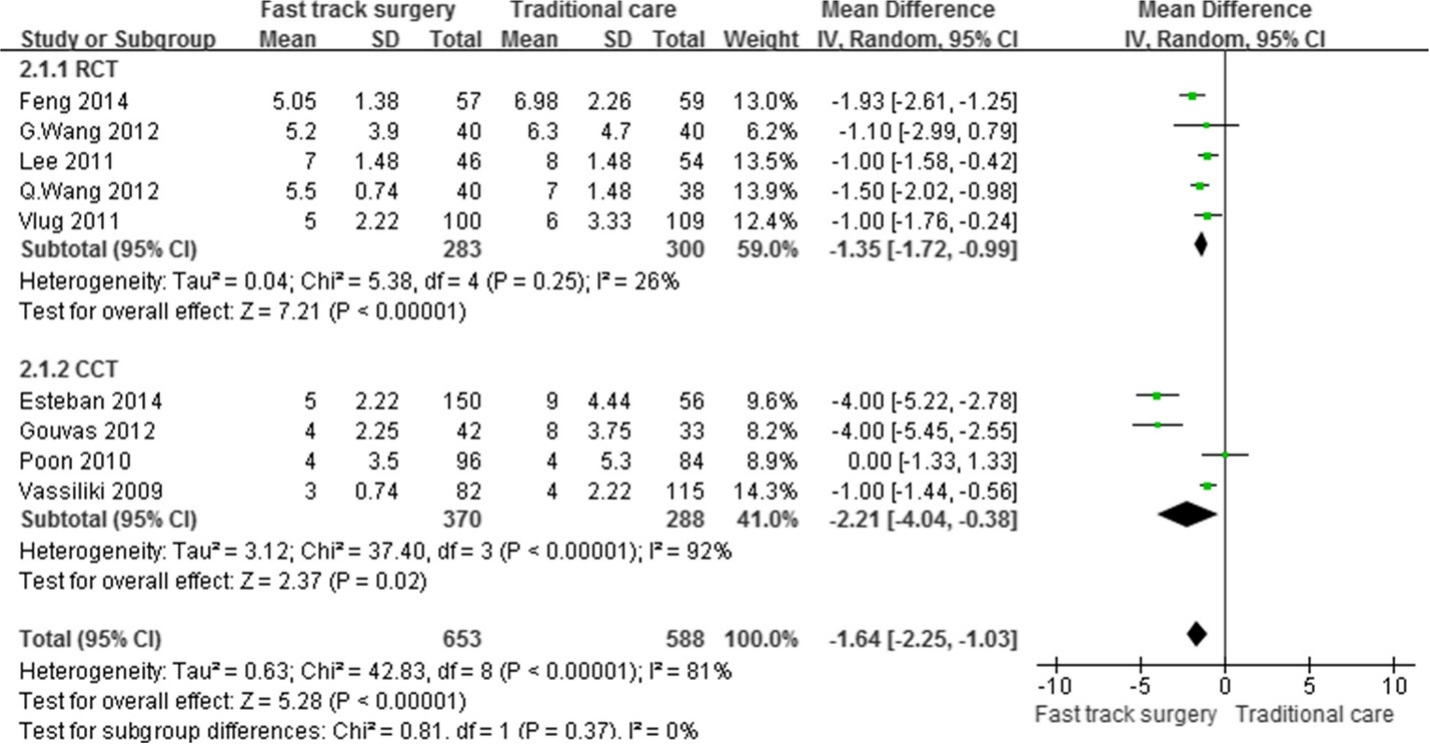
**Meta-analysis of postoperative hospital stay.**

All of the studies [16–25] reported the duration of the postoperative hospital stay. Notably, the outcome of the study by Huibers et al. [25] deviated significantly from the normal distribution. Thus, the outcome was not included in the meta-analysis. After pooling the data, there was a significantly shorter postoperative hospital stay favoring FTS (WMD, –1.64 days; 95% CI, –2.25 to –1.03; p < 0.001). The difference remained significant based on subgroup analysis of RCTs and CCTs. A random-effects model was used for significant heterogeneity between the studies (p < 0.001, I^2^ = 81%) (Figure 2).

### Time to first flatus

**Figure 3:**
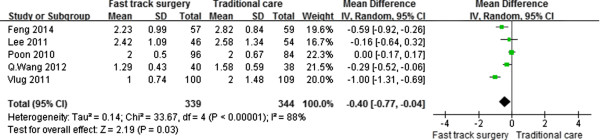
**Meta-analysis of time to first flatus.**

Five studies [16, 18–20, 22] reported the time to first flatus, which was significantly shorter in the FTS group than in the traditional care group (WMD, –0.40 day; 95% CI, –0.77 to –0.04; p = 0.03). A random-effects model was used for significant heterogeneity between studies (p < 0.001, I^2^ = 88%) (Figure 3).

### Time of first bowel movement

**Figure 4:**
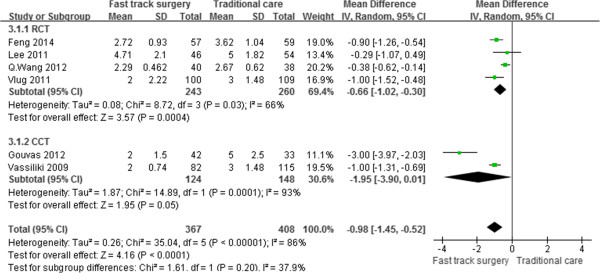
**Meta-analysis of first bowel movement time.**

Seven studies [16, 18–21, 24, 25] reported the time that elapsed until the first postoperative bowel movement. Notably, the outcome of the study by Huibers et al. [25] departed significantly from the normal distribution. Thus, the outcome was not included in the meta-analysis. After pooling the data, the time of the first bowel movement was significantly shorter in the FTS group than in the traditional care group (WMD, –0.98 day; 95% CI, –1.45 to –0.52; p < 0.001); however, the difference was not statistically significant based on the subgroup analysis of CCTs. A random-effects model was used for significant heterogeneity between studies (p < 0.001, I^2^ = 86%) (Figure 4).

### Total postoperative complication rate

**Figure 5:**
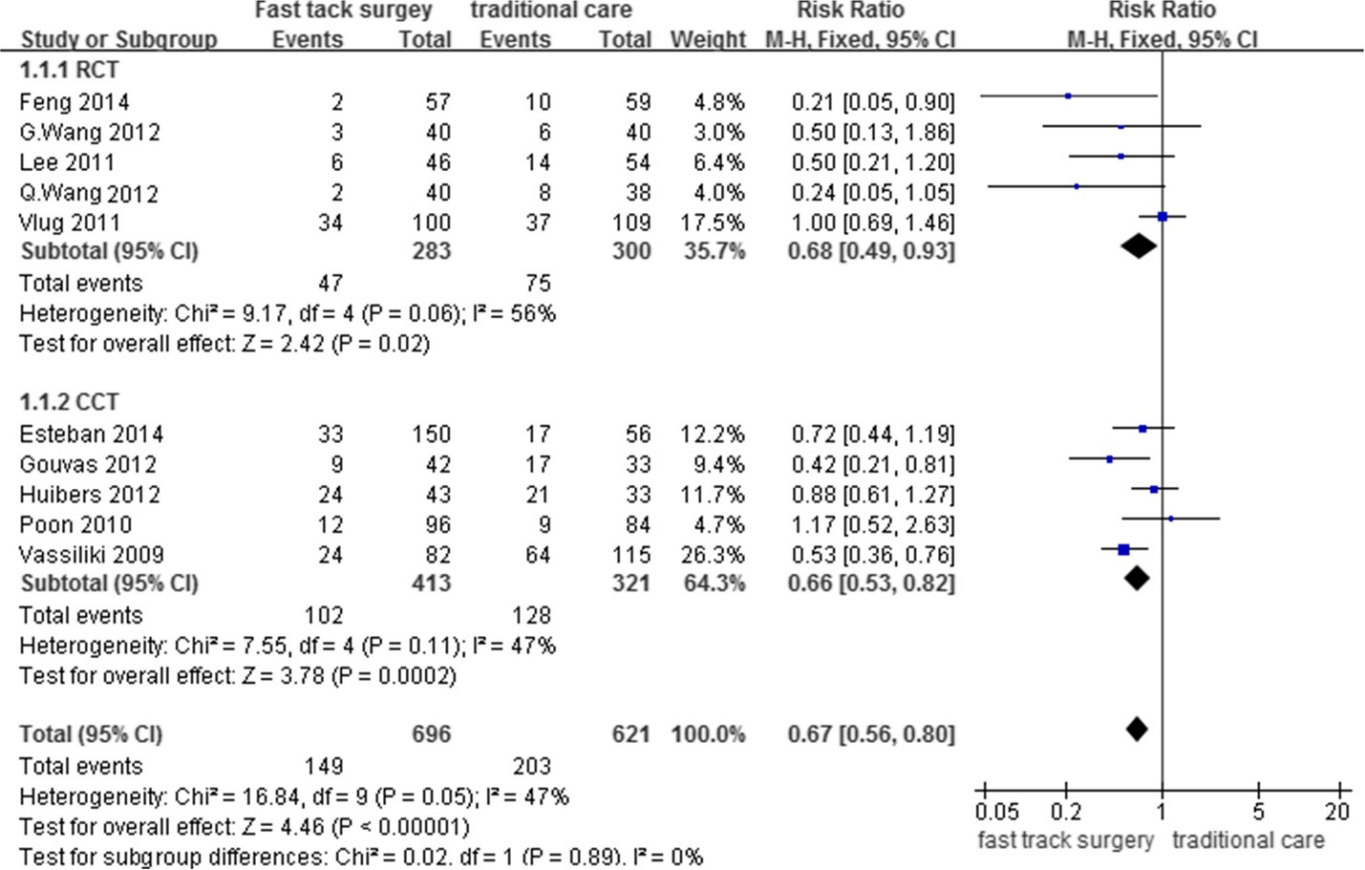
**Meta-analysis of total postoperative complication rate.**

All of the studies [16–25] reported the complication rate. A total of 149 patients in the FTS group developed complications, while 203 patients in the traditional care group developed complications. The results of the meta-analysis showed that FTS is associated with a significantly lower complication rate (RR, 0.67; 95% CI, 0.56–0.80; p < 0.001). Subgroup analysis of the RCTs and CCTs also showed a significant difference favoring FTS. There was no significant heterogeneity between studies (p = 0.05, I^2^ = 47%) (Figure 5).

### Rate of readmission

**Figure 6:**
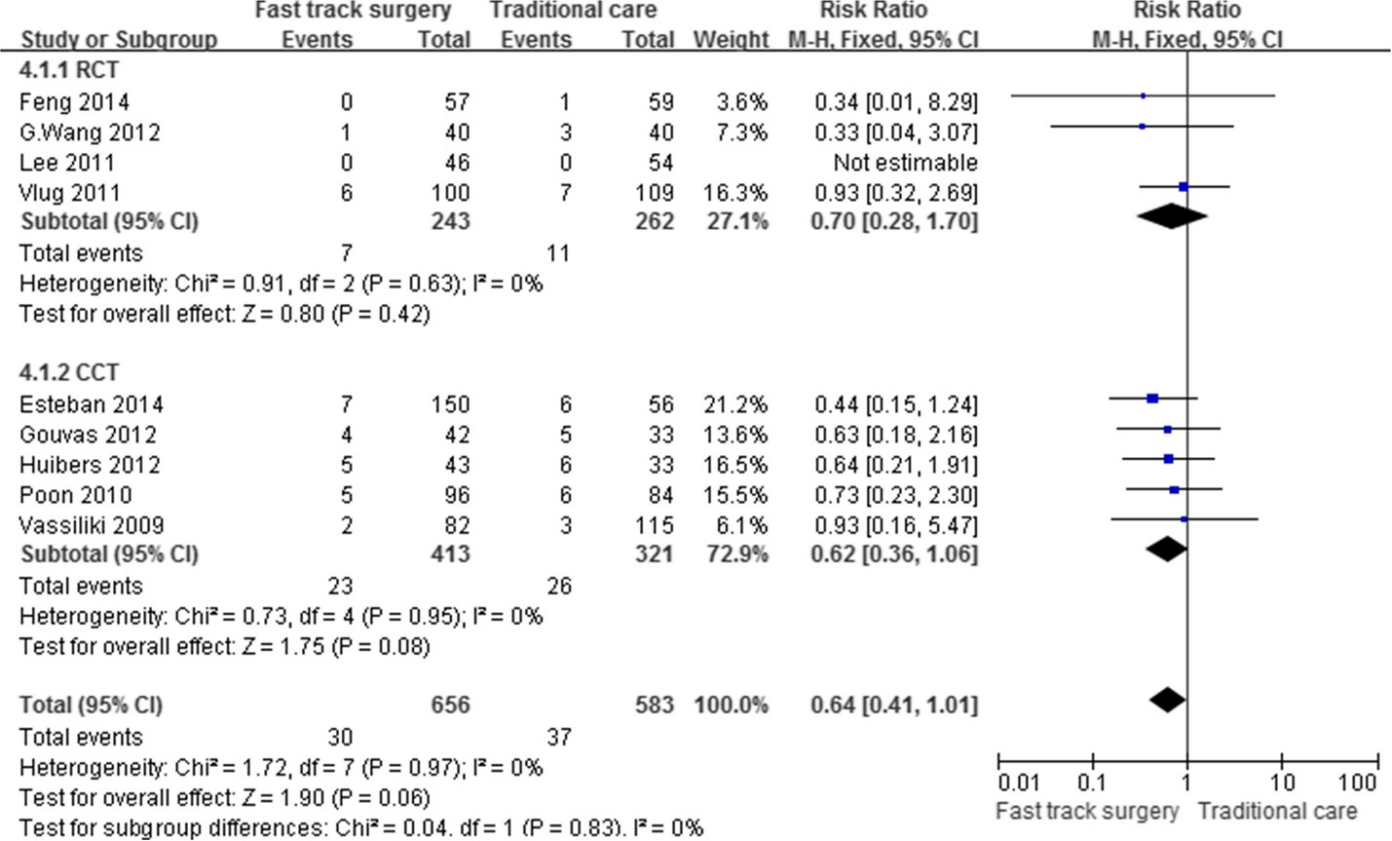
**Meta-analysis of the readmission rate.**

Nine [17–25] of the 10 studies reported the rate of readmission. Thirty patients in the FTS group and 37 patients in the traditional care group required readmission. Based on the meta-analysis, patients in the FTS group had a lower readmission rate; however, the difference was not significant (RR, 0.64; 95% CI, 0.41–1.01; p = 0.06). Additionally, subgroup analysis of RCTs and CCTs did not show a significant difference between the two groups. There was no significant heterogeneity between the studies (p = 0.97, I^2^ = 0%) (Figure 6).

### Thirty-day postoperative mortality

**Figure 7:**
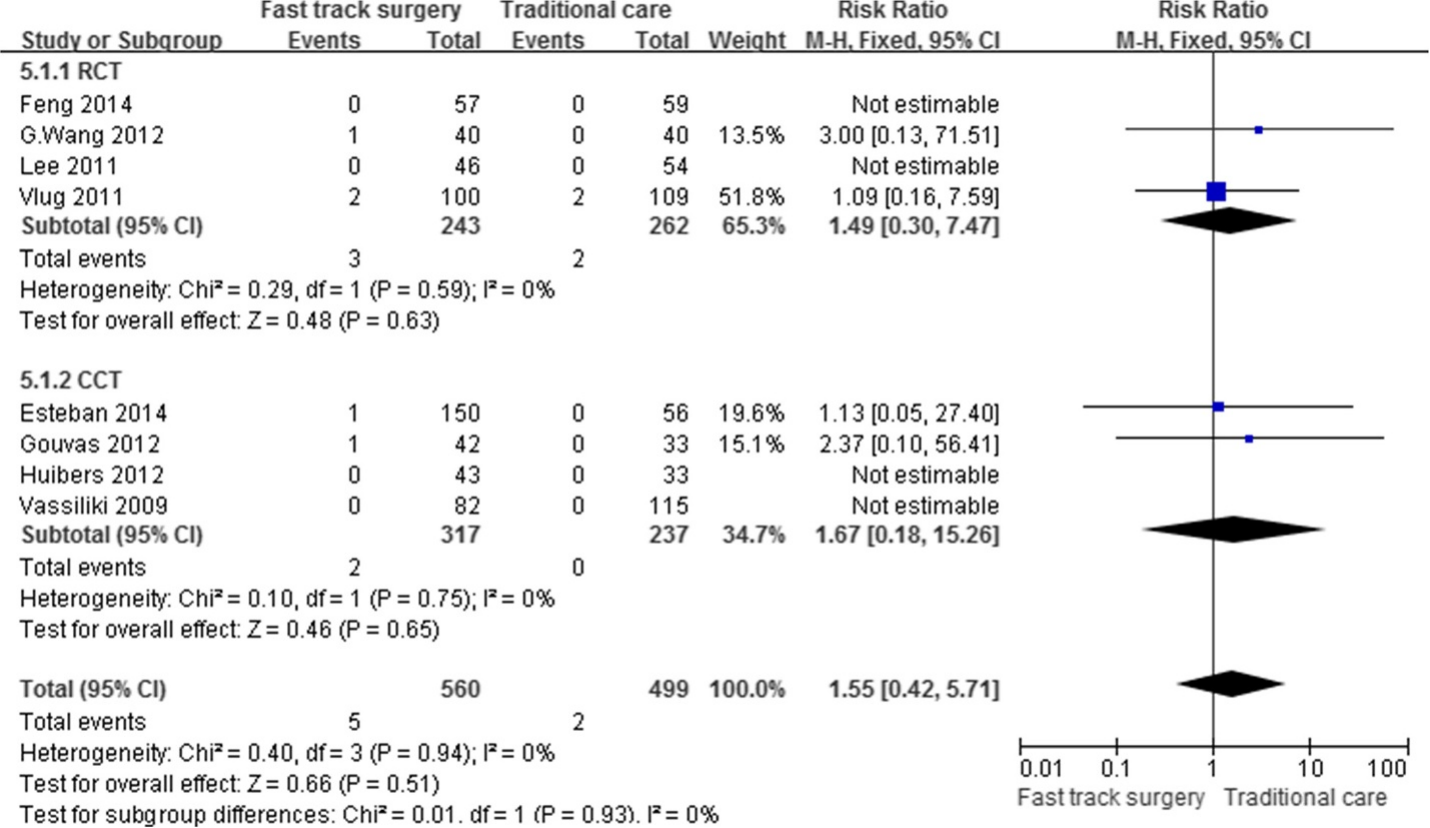
**Meta-analysis of thirty-day postoperative mortality.**

Eight [17–21, 23–25] of the 10 studies reported mortality rates. Five patients in the FTS group and two in the traditional group died 30 days after surgery. Based on the meta-analysis, no difference was present between the two groups (RR, 1.55; 95% CI, 0.42–5.71; p = 0.51). The subgroup analysis of RCTs and CCTs showed the same results as did the overall meta-analysis. There was no significant heterogeneity between the studies (p = 0.94, I^2^ = 0%) (Figure 7).

### Subgroup analysis based on fast-track elements

Subgroup analysis was performed based on each fast-track element for the duration of the postoperative hospital stay and total postoperative complication rate. For the duration of the postoperative hospital stay, the difference between the FTS group and traditional care group was not significant in the studies without the element “no bowel preparation”. For the total postoperative complication rate, the differences between the FTS group and traditional care group were not significant in the studies with the elements “no premedication”, “prevention of hypothermia”, “wound infiltration with local analgesia”, “minimally invasive incisions”, “no routine use of drains”, and “no morphine use”, separately. All other subgroup analysis results showed significant differences favoring FTS. The results are summarized in Table 5.

**Table 5:** **The results of subgroup analysis based on fast track elements**

Factor	OR for postoperative complication rate		WMD for postoperative hospital stay	
	Studies with the element	Studies without the element	Studies with the element	Studies without the element
A	0.69 (0.57-0.82), I^2^ = 43%	0.21 (0.05-0.90),*	-1.61 (-2.29,-0.92), I^2^ = 82%	-1.93 (-2.61,-1.25),*
B	0.70 (0.56-0.87), I^2^ = 50%	0.60 (0.44-0.83), I^2^ = 38%	-2.18 (-3.06,-1.31), I^2^ = 81%	-0.94 (-1.27,-0.60), I^2^ = 1%
C	0.67 (0.49-0.93), I^2^ = 52%	0.62 (0.40-0.95), I^2^ = 49%	-1.68 (-2.35,-1.01), I^2^ = 79%	**-1.61 (-3.52,0.29), I** ^**2**^ **= 89%**
D	**0.86 (0.66-1.13), I** ^**2**^ **= 43%**	0.57 (0.45-0.72), I^2^ = 12%	-1.34 (-1.77,-0.91), I^2^ = 11%	-1.81 (-2.67,-0.95), I^2^ = 86%
E	0.75 (0.61-0.92)**,** I^2^ = 34%	0.48 (0.33-0.69), I^2^ = 33%	-1.75 (-2.61,-0.88), I^2^ = 84%	-1.43 (-2.34,-0.52), I^2^ = 80%
F	0.69 (0.52-0.91), I^2^ = 48%	0.62 (0.41-0.94), I^2^ = 53%	-4.00 (-4.93,-3.07), I^2^ = 0%	-1.20 (-1.44,-0.95), I^2^ = 42%
G	**0.71 (0.50-1.02), I** ^**2**^ **= 55%**	0.56 (0.42-0.76), I^2^ = 13%	-2.63 (-3.98,-1.27), I^2^ = 88%	-1.10 (-1.38,-0.82), I^2^ = 23%
H	0.74 (0.60-0.93)**,** I^2^ = 42%	0.56 (0.41-0.76), I^2^ = 41%	-2.28 (-3.45,-1.10), I^2^ = 85%	-1.11 (-1.68,-0.55), I^2^ = 65%
I	**0.82 (0.41-1.63), I** ^**2**^ **= 56%**	0.58 (0.42-0.72), I^2^ = 27%	-1.10 (-2.09,-0.12), I^2^ = 74%	-1.96 (-2.80,-1.13), I^2^ = 86%
J	1.00 (0.69,1.46),*	0.59 (0.48,0.73), I^2^ = 37%	-1.00 (-1.76,-0.24),*	-1.74 (-2.43,-1.05), I^2^ = 83%
K	0.78 (0.63-0.96), I^2^ = 24%	0.45 (0.31-0.64), I^2^ = 19%	-1.82 (-2.99,-0.65), I^2^ = 87%	-1.43 (-1.95,-0.91), I^2^ = 64%
L	**0.81 (0.62-1.07), I** ^**2**^ **= 33%**	0.56 (0.44-0.71), I^2^ = 50%	-1.64 (-2.79,-0.49), I^2^ = 83%	-1.58 (-2.32,-0.84), I^2^ = 82%
M	-	-	-	-
N	-	-	-	-
O	**0.71 (0.46-1.08), I** ^**2**^ **= 69%**	0.60 (0.44-0.82), I^2^ = 17%	-1.29 (-1.88,-0.70), I^2^ = 64%	-1.90 (-2.95,-0.86), I^2^ = 86%
P	0.79 (0.63-0.99), I^2^ = 27%	0.53 (0.40-0.71), I^2^ = 30%	-1.74 (-2.67,-0.81), I^2^ = 85%	-1.58 (-2.85,-0.58), I^2^ = 82%
Q	0.69 (0.57-0.83), I^2^ = 44%	0.42 (0.21-0.81),*	-1.42 (-1.97,-0.87), I^2^ = 77%	-4.00 (-5.45,-2.55),*

A: patients education B: preoperative feeding C: No bowel preparation D: No premedication E: fluid restriction F: high O2 concentration during operation G: prevention of hypothermia H: epidural analgesia I: wound infiltration with local analgesia J: minimally invasive incisions K: No routine use of NG tube L: No routine use of drains M:early mobilization N:enforced early postoperative oral feeding O: No morphine use P: standard laxatives Q: early remove bladder catheter. “*” only one study in the subgroup, no I^2^ could be provided. “-“ all the including studies contain the element. The results without significant difference is marked by bold type.

### Other outcomes

Data on some other outcomes were impossible to subject to meta-analysis because of incompatibility or the limited study quantity. Thus, we performed a systemic review. Pain control or pain intensity after surgery was reported in four studies [18–21], three of which [19–21] showed significantly less pain in patients who underwent FTS. Moreover, Wang et al. [16] included the serum parameters after surgery. The C-reactive protein and interleukin-6 levels were significantly lower in the FTS group. Additionally, the quality of life after surgery and in-hospital costs were reported by one [18] and two studies [18, 20], respectively. Vlug et al. [18] showed no significant differences in these outcomes between the two groups; however, Feng et al. [20] showed that FTS was associated with significantly lower medical costs.

## Discussion

Over the past 20 years, FTS and laparoscopic techniques have become the two primary methods of reducing surgical stress and improving recovery after colorectal surgery, thus providing better short-term outcomes. Combining the two approaches would hypothetically result in the most rapid recovery. Thus, we conducted the present study to provide evidence in support of this theory. Our results suggest that both the postoperative hospital stay, time to first bowel movement and the time to first flatus were shorter in the FTS group than in the traditional care group after laparoscopic colorectal surgery. Two recent meta-analyses [11, 26] that compared FTS with traditional care for all types of colorectal surgery suggested that hospital stays were shorter in the FTS group, which is in agreement with our findings. Furthermore, because both FTS and laparoscopy can reduce surgical stress and improve recovery, incorporation of FTS into laparoscopic surgery is not superfluous and may have a combined effect in enhancing recovery and shortening the postoperative hospital stay.

Safety is always of utmost concern in clinical practice. Although reducing the complication rate is one of the aims of FTS, concerns have been expressed about the increased risk of severe complications such as pulmonary embolism and anastomotic leakage [27]. Previous meta-analysis of FTS in all types of colorectal surgery suggested that FTS neither compromise nor enhance safety [11, 26]. However, our results suggest that FTS is associated with a significantly lower complication rate than traditional care is. This is a surprising result. First, this finding may have been caused by the adequate fast-track elements in the included studies. Second, this result may have been associated with the combined effect of laparoscopic techniques and FTS with available expertise of the medical team [2, 28]. Another concern about FTS is the potentially higher readmission rate reported by some hospitals [29]. After pooling the data, FTS was associated with a relatively lower readmission rate. This finding may be attributed to the rigid and strict discharge criteria in the FTS protocols of the included studies [11]. Based on our results, we can conclude that FTS is feasible and can enhance safety after laparoscopic colorectal cancer surgery. Adequate fast-track elements and rigid and strict discharge criteria are two important factors that contribute to this conclusion.

As mentioned above, adequate fast-track elements applied in the included studies were an important prerequisite for the encouraging results. This is also why we excluded studies with fewer than six fast-track elements. We did not include the study by Chalabi et al. [30] because they applied a “RAPID protocol”, which is a simplified fast-track protocol that contains only three fast-track elements. However, distinctions among the fast-track elements were not preventable among the included studies. This may also explain the heterogeneity in some outcome measures.

Thus, to provide better evidence, we performed a subgroup analysis based on each fast-track element for two major outcomes: the duration of the postoperative hospital stay and the total postoperative complication rate, each of which can separately represent the efficacy and safety of FTS. Our results indicate the importance of the fast-track element “no bowel preparation” because the difference in the duration of the postoperative hospital stay between the FTS and traditional care group was not significant in the studies without the element “no bowel preparation”. Two comprehensive studies also suggested that bowel preparation is unnecessary [31, 32]. Several RCTs showed that bowel preparation was associated with a prolonged hospital stay and higher complication rate [33, 34]. Therefore, “no bowel preparation” should be a priority when establishing a fast-track protocol in the future. Additionally, differences in the total postoperative complication rate between the FTS and traditional care group were not significant in the subgroup analysis of many elements. Notably, subgroup analysis results of the element “wound infiltration with local analgesia” deviated greatly from statistical significance (OR, 0.82 [0.41–1.63]; p = 0.57). At the same time, the effect of local infiltration analgesia is questionable [35]. RCTs and meta-analysis on this topic have also shown controversial results [36–38]. Therefore, we do not recommend integration of the element “wound infiltration with local analgesia” into FTS. More high-quality RCTs are required to provide more solid evidence regarding this element.

Another issue regarding the fast-track elements is that no presented FTS guidelines are particular for laparoscopic surgery, and some useful fast-track elements are debatable in laparoscopic surgery. In particular, epidural analgesia has been proven to provide better pain relief, reduce perioperative stress, reduce postoperative complications, and shorten the hospital stay after open surgery [6, 39]; however, its role in laparoscopic surgery remains controversial. On one hand, six studies used epidural analgesia, which showed wide acceptance. The beneficial effect of epidural analgesia in pain control has also been confirmed by many studies [40, 41]. On the other hand, epidural analgesia during laparoscopic surgery is not advocated by some authors. The meta-analysis conducted by Levy et al. [40] suggested that no analgesia protocol showed more overall benefits than did other protocols during laparoscopic surgery. Another meta-analysis showed that epidural analgesia fails to shorten the hospital stay following laparoscopic colorectal surgery [41]. Moreover, even Kehlet [2], who initiated FTS, demonstrated that epidural analgesia might not be necessary in laparoscopic colorectal surgery and can be replaced by non-opioid analgesia. Given the limited number of studies in this specific clinical area, more evidence is required to determine the role of epidural analgesia in the fast-track protocol for laparoscopic colorectal surgery.

Patient selection is also a debatable issue in FTS. Feroci et al. [42] suggested that patients >75 years of age with an American Society of Anesthesiologists (ASA) physical status score of 3 or 4 have high complication rates, prolonged hospital stays, and negative compliance. Male sex is another predictor of negative compliance. Among the included studies, the baseline characteristics were comparable between the FTS and control groups in the studies published by Poon et al. [22], Vassiliki et al. [24], and all RCTs. Compared with the traditional care group, Gouvas et al. [21] enrolled more male patients, Esteban et al. [23] enrolled more patients with high ASA scores, and Huibers et al. [25] enrolled more patients with advanced-stage tumors in the FTS group. Male sex, a high ASA score, and advanced-stage tumors were factors associated with poor outcomes. Thus, the effect of FTS may have been more significant without these baseline differences. The differences in patient selection among the different studies is another issue. Wang et al. [16] focused on elderly patients with a higher mean age than in other studies. Vassiliki et al. [24] enrolled more patients with ASA scores of 3 and 4. The ratio of patients with advanced-stage tumors in the study by Poon et al. [22] was also relatively higher than in other studies. Although all of these studies showed results favoring FTS, the above-mentioned differences may be another factor that contributed to the heterogeneity.

**Figure 8:**
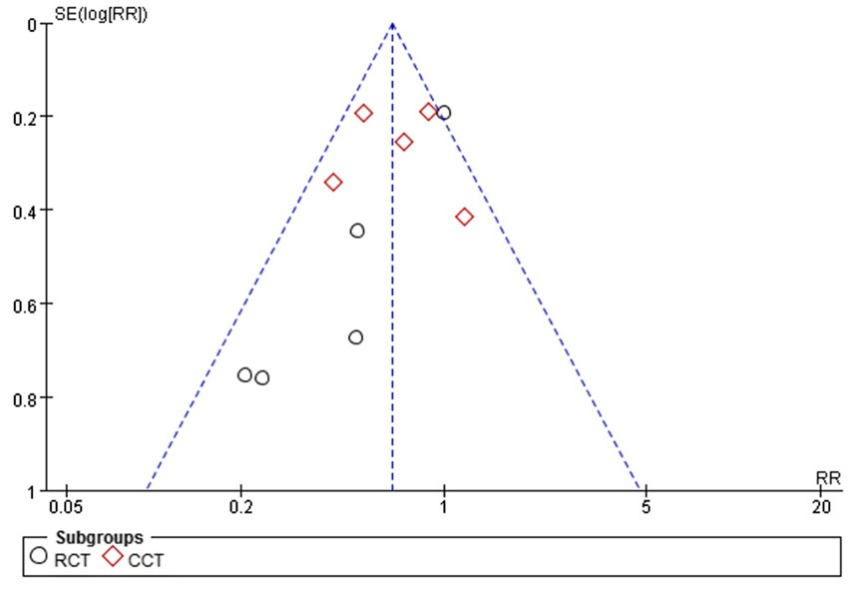
**Funnel plot of the studies on the rate of postoperative complications.**

A previous meta-analysis [43] was conducted on this topic. In contrast to their study, we included CCTs and one new RCT [20]. We also excluded three RCTs [44–46] that were included in the above-mentioned meta-analysis by mistake. Most importantly, we excluded the study by Wang [44] because in that study, FTS and traditional care were compared in all types of colorectal surgery, not only in laparoscopic surgery. We also excluded the studies by van Bree [45] and Veenhof [46] because they exhibited overlap of patients and authors with the study by Vlug [18]. Thus, we suppose that our study provides better evidence.Several limitations of this meta-analysis should be considered. First, some variables such as the skill and experience of the operating surgeon, efficacy of perioperative care, and quality of anesthesia may have differed between the FTS and traditional care groups. Thus, further high-quality, large-scale, and multicenter RCTs should be performed with consideration of these differences between the two groups. Second, 5 of 10 studies were not RCTs, which may have compromised the statistical power. Third, the surgery type varied among the studies, and subgroup analysis was not performed because of unextractable data. Finally, as mentioned above, considerable heterogeneity was observed in our study. Despite of these limitations, our meta-analysis shows some favorable results and conclusions regarding the effects of FTS after laparoscopic colorectal surgery. In particular, we found that FTS can enhance safety. At the same time, no obvious publication bias was observed by performing a funnel plot on the rate of postoperative complications (Figure 8).

## Conclusion

In laparoscopic colorectal cancer surgery, FTS can significantly shorten the postoperative hospital stay, accelerate the postoperative recovery, and, notably, enhance safety when compared with traditional care. In the future, more high-quality and well-designed studies are needed to provide more solid evidence.

## Abbreviations

FTS: Fast-track surgery
RCTs: Randomized controlled trials
FT: Fast track
CCTs: Controlled clinical trials
WMD: Weighted mean difference
CIs: Confidence intervals
SD: Standard deviations
IQR: Interquartile range.
